# Wearable Microfluidic Sweat Chip for Detection of Sweat Glucose and pH in Long-Distance Running Exercise

**DOI:** 10.3390/bios13020157

**Published:** 2023-01-19

**Authors:** Dong Liu, Zhenyu Liu, Shilun Feng, Zehang Gao, Ran Chen, Gaozhe Cai, Shengtai Bian

**Affiliations:** 1 Microfluidics Research & Innovation Laboratory, School of Sport Science, Beijing Sport University, Beijing 100084, China; 2 School of Kinesiology, Shanghai University of Sport, Shanghai 200438, China; 3 State Key Laboratory of Transducer Technology, Shanghai Institute of Microsystem and Information Technology, Chinese Academy of Sciences, Shanghai 200050, China; 4 Department of Clinical Laboratory, Third Affiliated Hospital of Guangzhou Medical University, Guangzhou 510150, China; 5 School of Management, Beijing Sport University, Beijing 100084, China

**Keywords:** exercise monitoring, microfluidics, sweat analysis, glucose, pH

## Abstract

Traditional exercise training monitoring is based on invasive blood testing methods. As sweat can reveal abundant blood-related physiological information about health, wearable sweat sensors have received significant research attention and become increasingly popular in the field of exercise training monitoring. However, most of these sensors are used to measure physical indicators such as heart rate, blood pressure, respiration, etc., demanding a versatile sensor that can detect relevant biochemical indicators in body fluids. In this work, we proposed a wearable microfluidic sweat chip combined with smartphone image processing to realize non-invasive in situ analysis of epidermal sweat for sports practitioners. The polydimethylsiloxane (PDMS) based chip was modified with nonionic surfactants to ensure good hydrophilicity for the automatic collection of sweat. Besides, a simple, reliable, and low-cost paper-based sensor was prepared for high-performance sensing of glucose concentration and pH in sweat. Under optimized conditions, this proposed chip can detect glucose with low concentrations from 0.05 mM to 0.40 mM, with a pH range of 4.0 to 6.5 for human sweat. The ability of this microfluidic chip for human sweat analysis was demonstrated by dynamically tracking the changes in glucose concentration and pH in long-distance running subjects.

## 1. Introduction

In traditional exercise monitoring, blood has been considered the gold-standard body fluid [1,2,3,4,5]. However, blood testing is always accompanied by an invasive sampling process, which causes a certain degree of pain that subjects could not accept. With the development of technology, some non-invasive body fluids, such as sweat, tears, interstitials, and saliva, can indicate the physical state of the body and have attracted much attention for exercise monitoring [6,7]. Due to their non-invasive sample harvesting methods being more suitable for real-time exercise monitoring, those body fluids have become a new alternative to blood. An enzymatic glucose sensor-based contact lens has been successfully developed for electrochemical monitoring of glucose concentration in tears [8]. The contact lens holds excellent potential in continuous glucose management. Besides, bioinformatic substances such as hormones (e.g., cortisol), protein (e.g., IL-8), and antibodies (e.g., HIV) have been successfully identified in human saliva samples [9]. It can be seen that the human body fluid contains a considerable amount of biological information, which can be used as a valuable biomarker for exercise monitoring.

Sweat is a readily accessible biofluid that can be collected non-invasively as well. When the body temperature rises due to heavy exercise, electrolytes and liquid are excreted by sweat glands, which are mainly composed of secretory cells and myoepithelial cells, via an elongated duct to the outer skin surface [10,11]. In the sweat of the secretion, many substances related to the physiological state of the human body, including inorganic, metabolites, acids, hormones, and small proteins/peptides, passively or actively penetrate sweat from nearby blood or interstitial fluids [12,13]. Glucose is the most critical energy supply of the body, and the fluctuation of glucose level will affect performance during exercises [14,15]. Due to the highly vascularized structure of eccrine glands and the osmotic pressure-driven way of sweat secretion, there is a certain correlation between sweat glucose and blood glucose [16]. As a result, sweat has emerged as a promising alternative to blood samples for the painless evaluation of glucose levels in the body.

Similarly, the pH value of body fluid is closely related to homeostasis [17,18]. The pH in sweat is also a key factor for exercise monitoring that is closely related to electrolyte concentration. It was reported that the pH value in sweat during exercise would change due to the onset of metabolic alkalosis [19]. For example, the pH value of a healthy person is in the range of 4.5–6.5 [10,20]. During exercise, the concentration of ammonia in sweat decreases due to its conversion to ammonium (NH_4_^+^). Since NH_4_^+^ diffuses less across cellular membranes than ammonia, the excess NH_4_^+^ accumulates in large quantities, leading to an increase in the pH value in sweat [20]. It means that the measurements of pH in sweat could be used to correlate the build-up of acid within muscle cells while exercising, which leads to muscle fatigue. In addition, the pH value in sweat is also an indicator of the body’s exercise intensity and degree of dehydration.

In recent years, wearable sweat analysis devices have emerged for non-invasive sampling and in situ sweat analysis. Electrochemical detection is one of the commonly used methods in sweat analysis, it can effectively convert the concentration of analytes into electrical signals. Sweat analysis equipment based on electrochemical detection technology relies on powerful integrated circuit manufacturing technology and can achieve multi-channel accurate detection in sweat analysis [20,21,22,23]. Changing the different electrochemical detection modules can accurately detect the concentration of lactic acid, glucose, electrolytes, caffeine, and pH in sweat. However, it is not suitable for sweat analysis in exercise scenes with many interference factors as the signal is susceptible to interference due to the complex structure of electrochemical sweat analysis equipment. Another sweat analysis device based on colorimetric detection technology is more suitable for sweat analysis in exercise due to its simple structure and easy-to-read signals [24,25,26,27,28,29,30]. A polydimethylsiloxane (PDMS)-based stretchable microfluidic device was fabricated to route sweat into chambers containing colorimetric assay reagents for the measurements of metabolites [24,28,29,30] and sweating rate [25,26]. To realize the sweat analysis of water sports, a waterproof wearable sweat epidermal patch was designed [27]. The patch combined a colorimetric sensing system with an NFC electronic module encapsulated in a waterproof coating, attached to the skin using a water-resistant adhesive layer with low water permeability, and it can enable the capture, storage, and analysis of sweat even fully underwater. Although colorimetric sensing technology has been developed maturely, there is still a need for a simple, convenient and low-cost colorimetric sweat detection chip to meet the needs of high reliability and mass production in sports scenarios.

**Figure 1:**
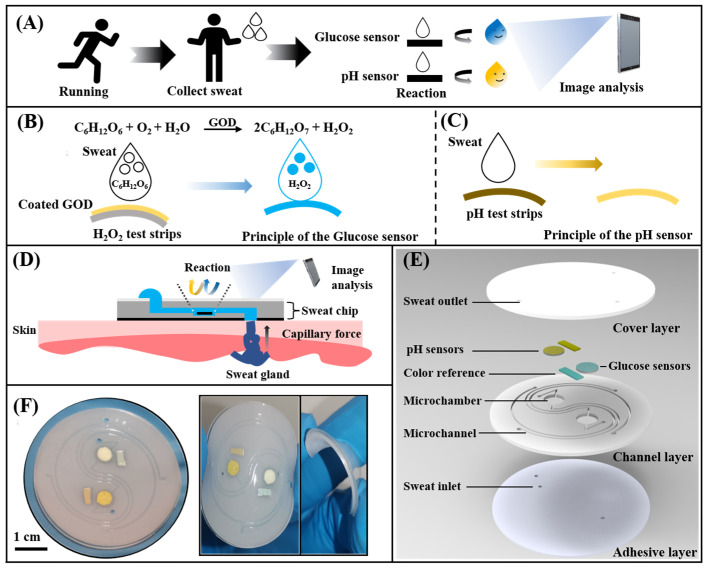
Schematic and optical images of the microfluidic sweat chip. (**A**) Conceptual diagram of the microfluidic sweat chip. (**B**,**C**) Principle of the glucose sensor and pH sensor. (**D**) Schematic of the sweat collection using the microfluidic sweat chip. (**E**) Schematic diagram of the microfluidic sweat chip structure. (**F**) Image of the microfluidic sweat chip.

Here, we proposed a wearable microfluidic chip based on a non-invasive instant colorimetric to realize the detection of glucose concentration and pH value in the sweat of long-distance runners. The wearable microfluidic chip can collect body surface sweat at any moment during the exercise (Figure 1A). Sweat was rapidly drawn into detection chambers by capillary force through Triton X-100 modified hydrophilic microchannels. Glucose concentration and sweat pH were converted into color signals by a glucose sensor and a pH sensor. Then, a colorimetric detection method based on a smartphone was used to acquire optical images of color and quantitatively analyze the glucose and the pH value (Figure 1C). The chip consists of a hydrophilic PDMS layer with channels and chambers, a smooth PDMS cover layer, a colorimetric sensing system, and a bio-adhesive layer (Figure 1D). Electrochemical detection techniques are often used in previous glucose sensing systems [31] Such electrochemical systems involves the use of copper oxide [32], multi-walled carbon nanotubes [33], and highly efficient catalysts [34] to improve the accurate detection of glucose. In addition, colorimetric detection techniques based on the TMB-HRP enzymatic system are also commonly used in glucose sensing systems [28,35,36], and the detection of pH value was usually completed by an electrochemical detection module [20,22,23]. In these methods, there were strict restrictions on manufacturing and use conditions. We presented a simple and accurate paper-based sensor based on rapid detection test strips to adapt to the demand for simple, efficient, and low-cost colorimetric sensing. Glucose oxidase (GOD) could oxidize glucose in sweat to generate hydrogen peroxide, which could be detected by a commercial test strip to quantify the concentration of glucose. The detection of pH value was performed by directly acquiring the color signal of the pH test strip (Figure 1B). The proposed chip can achieve glucose detection with concentrations from 0.05 mM to 0.40 mM and a pH range of 4.0 to 6.5 in sweat. Finally, by detecting the glucose concentration in the sweat of long-distance runners at different times, comparing the changes of sweat glucose and blood glucose during exercise and the effect of ingesting sports supplements on sweat glucose and pH value, the capability of this chip to non-invasively monitor human sweat in situ during exercise was demonstrated.

## 2. Materials and Methods

### 2.1. Materials and Reagents

The Triton X-100 (99.0%), HCl (37.0%), NaOH (0.10 mM), artificial sweat, glucose oxidase (GOD), and phosphate buffered saline (PBS) were purchased from Sigma-Aldrich (St. Louis, MO, USA). Glucose was purchased from Aladdin Reagent Co., Ltd. (Shanghai, China). The silicone elastomer kit for fabricating the polydimethylsiloxane (PDMS) chip was purchased from Dow Corning (Sylgard 184, Auburn, MI, USA). Hydrogen peroxide test strips (1.0–100.0 mg), pH value test paper (0–14.0), and standard colorimetric card (GSB05-1426-2001) were all obtained from Lohan Environment Technology Co., Ltd. (Hangzhou, Zhejiang, China). Deionized (DI) water was generated by a Millipore water purification system. All other reagents were of analytical grade and directly used without further purifications.

### 2.2. Preparation of the Microfluidic Chip

The microfluidic chip includes a PDMS cover layer and a PDMS channel layer with microfluidic channels and chambers, and a waterproof medical biological double-sided adhesive pasted under the PDMS channel layer. Figures S1 and S2 show the details of the fabrication processing of microfluidic chips and the detailed chip design parameters. The mold of the PDMS channel layer was fabricated by patterning negative photoresist (SU-8 3050, Microchem, Newton, MA, USA) on a silicon wafer using a direct laser writing system (MicroWriter ML3, Durham Magneto Optics Ltd., London, UK). The SU-8 spin speed and time: 1000 rpm, 40 s; select exposure dose was 2200 mJ/cm^2^. The surface of PDMS was flattened by standing for 1 h on the horizontal plane. There are two reaction chambers with a volume of about 2.5 µL (diameter: 4 mm, height: 200 μm) and short straight ducts (width: 300 μm, height: 200 μm) on the PDMS layer. The PDMS prepolymer and curing agent were prepared in a mass ratio of 15:1, and 10% Triton X-100 was added to mix evenly, poured onto the fabricated mold followed by vacuuming for 30 min to remove the air between micropillars, and then cured at 80 °C for 1 h. After lifting the PDMS channel layer from the silicon mold, the inlets and outlets of the microchannel were punched using a punch with a diameter of 1.5 mm. The PDMS cover layer without Triton X-100 (thickness: 800 μm) was processed in the same way. The processed PDMS channel layer and PDMS cover layer were cut into circles (diameter: 30 mm) with a scriber. Then, the glucose sensor and the pH sensor were placed in the chamber of the PDMS channel layer, followed by bonding two PDMS layers together using oxygen plasma treatment (PDC-002, Harrick Plasma, Ithaca, NY, USA). Finally, three sweat inlets with a diameter of 3 mm were punched on the waterproof medical double-sided glue to ensure that each inlet could cover about 5 to 10 sweat glands. The microfluidic chip and the adhesive layer were aligned according to the position of the inlet and then bonded. The fabricated microfluidic sweat chip was placed in a plastic bag, vacuumed, and stored in a cool and ventilated place for subsequent use.

### 2.3. Preparation of Paper-Based Glucose and pH Sensors

The colorimetric sensor is a prominent part of the chip. Since that glucose is an important component of human sweat and sweat pH can be closely related to muscle fatigue, we chose to build a sensing device for in-situ sweat glucose concentration and pH monitoring. Firstly, 5.0 μL of the PBS solution (pH = 5.7) was dropped onto the H_2_O_2_ test strip. After drying at room temperature (25 °C) for 10 min, 5.0 μL of the GOD solution (200 U/mL) was then deposited on functionalized test strips to create a highly sensitive colorimetric glucose sensor. The pH sensor was formed by cutting the pH test paper according to the size of the chamber. The detailed manufacturing process could be found in Figure S3.

### 2.4. The Wearable Microfluidic Sweat Chip for Sweat Analysis

In this work, the PDMS-based microfluidic channel layer was used to collect and store sweat. Functionalized paper-based sensors were placed in two separate detection chambers to detect sweat glucose and pH value. The independent detection chamber can effectively avoid the cross-contamination of sweat samples and the irritation of the skin by reagents. The chip can be pasted on the forehead, chest, and arm of the human body (Figure S4), and collect the sweat produced during exercise. The glucose concentration and pH value in the sweat will be converted into optical color signals. Images were captured using a smartphone and color analysis was performed by the application software to determine the glucose concentration and pH in sweat samples. To avoid the influence of ambient light sources, the standard colorimetric card was cut and placed in the reference chamber.

### 2.5. Detection of Glucose and pH in Artificial Sweat

The artificial sweat was selected according to the standard ISO 3160-2 and glucose was added to it to obtain glucose samples with different concentrations. The pH value of artificial sweat was adjusted using HCl (37.0%) and NaOH (0.10 mM). The microfluidic sweat chip could inhale 5.0 μL of glucose samples with different concentration gradients and 5.0 μL of sweat samples with different pH values. A series of images of optical signals produced by the chip were acquired using a smartphone. The RGB values of the images were analyzed with Color Analysis software to obtain the color model and detection range of sweat glucose concentration and pH value.

### 2.6. Analysis of Epidermal Sweat in Long-Distance Running Subjects

Healthy young adults were chosen as the testing subjects to perform long-distance running in 30 min. The microfluidic sweat chip was attached to the inner side of the arm of the testing persons for the analysis of sweat. The concentration of the glucose in sweat and blood (the blood glucose concentration was collected by a blood glucose meter), as well as the sweat pH value of each subject, were detected at the time points of 10 min, 20 min, and 30 min during running. Subjects were supplemented with an alkaline soda water sports drink containing 50.0 g glucose immediately after exercising and rested for 1 h. Then, subjects ran again for 30 min to collect the sweat glucose, blood glucose concentration, and sweat pH in the same way.

## 3. Results and Discussion

### 3.1. Surface Hydrophilic Modification of Microfluidic Sweat Chip

**Figure 2:**
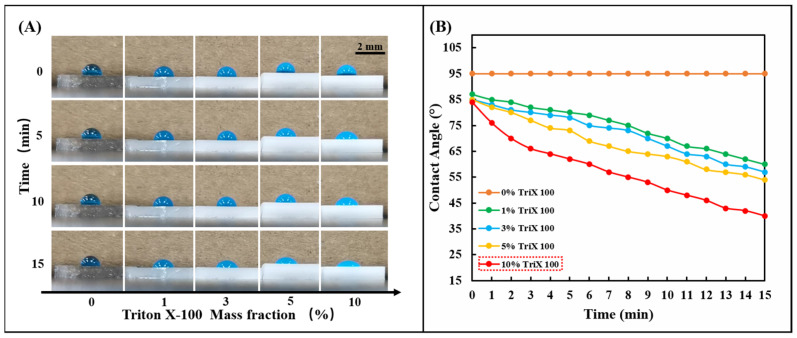
Hydrophilic treatment of PDMS with TritonX-100. (**A**) Change the mass percentage of Triton X-100 in PDMS (0, 1, 3, 5, 10%) and observe the change of the hydrophilic angle on the PDMS surface within 15 min. (**B**) The linear change rule of the hydrophilic angle on the surface of PDMS containing different concentrations of Triton X-100 (0, 1, 3, 5, 10%) within 15 min.

The silicone polymer PDMS used in the chip is naturally hydrophobic, and its inherent hydrophobic angle (CA) is between 95° and 107° [37]. Although sweat glands generate positive pressure during perspiration, the resistance created by the hydrophobic PDMS prevents sweat from entering the chip. Therefore, the hydrophilic performance of the PDMS channel layer is crucial to the smooth entry of sweat. This work used the nonionic surfactant Triton X-100 to modify the silicone polymer PDMS. Fabrication of hydrophilic microfluidic channels with negative capillary pressure using the modified PDMS can spontaneously draw sweat into the channels. A series of 1, 3, 5, and 10% Triton X-100 were mixed with PDMS (Prepolymer: Curing agent = 15:1). After fully stirring and curing at 85 °C for 30 min, 5.0 μL of blue ink was added dropwise to observe the change of the hydrophobic angle (CA) of the droplet (Figure 2A). It can be observed that the droplet contact angle (CA) decreases significantly on PDMS modified with nonionic surfactant over a period, which indicates that the nonionic surfactant (Triton X-100) in PDMS. The presence of ions significantly affects the interfacial surface tension and enhances the wettability of water. The wettability of the modified PDMS can be controlled by changing the initial concentration of Triton X-100 in the PDMS. By comparing the effects of different initial concentrations of Triton X-100 on the wettability of the PDMS surface (Figure 2B), it can be seen that Triton X-100 with a mass fraction of 10% has the most significant effect on the hydrophilic angle. In this study, 10% Triton X-100 was used to modify PDMS to ensure the hydrophilicity of the PDMS channel.

### 3.2. Selection of Color Model for Optical Image

**Figure 3:**
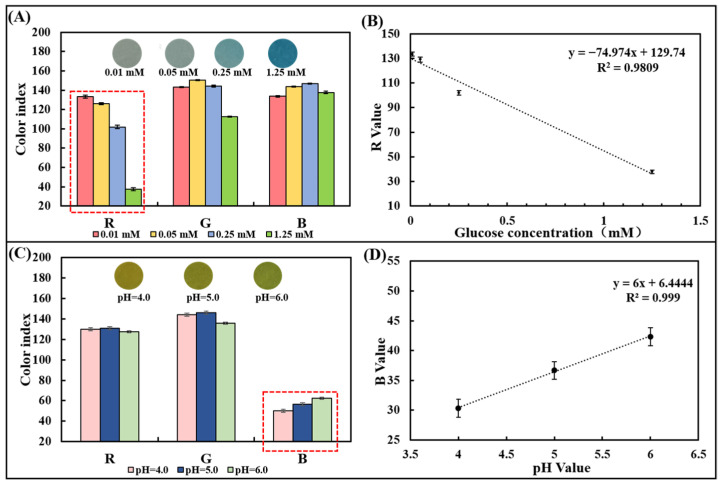
Optical color model selection for glucose concentration and pH detection using pure sample out of the chip. (**A**) RGB values of the paper-based glucose sensor after reaction with pure samples containing glucose with different concentrations. (**B**) Linear plot of the R-value obtained from the paper-based sensor versus the corresponding glucose concentration. (**C**) RGB values of the paper-based pH sensor after reaction with pure samples with different pH values. (**D**) The plot of the linear relationship between the R-value obtained from the paper-based sensor and the corresponding pH value.

A series of pure samples containing known glucose concentrations (0–1.25 mM) were dropped onto the glucose colorimetric sensor to trigger an enzyme-catalyzed color change. The optical image gradually appears from light blue to dark blue with the glucose concentration increasing (Figure 3A). To select a suitable optical color model, we designed and developed a smartphone-based image detection software through which optical images were collected, and the R, G, and B values in the image color were quantified. After measuring samples containing 0.01 mM, 0.05 mM, 0.25 mM, and 1.25 mM glucose, the differences in R, G, and B values were analyzed comparatively. It was found that there was a linear relationship between the R-value (y) and the glucose concentration (x) from 0.01 mM to 1.25 mM (Figure 3A,B), which can be expressed as y = −74.974x + 129.74 (R^2^ = 0.9809). Thus, the R-value of the image was chosen as the signal for the detection of glucose in the chip. 

Similarly, the pH colorimetric sensor was dropped with a series of pure samples of known pH (4.0–6.0). The optical image gradually changes from yellow to yellow-green (Figure 3C). After measuring the pH = 4.0, 5.0, and 6.0 samples, the R, G, and B values were compared and analyzed. Since differences in gradients can be observed in the B value, it was chosen as the readout to prepare the calibration curve. A positive linear relationship between the B value and pH value between 4.0 and 6.0 could be observed and the linearity is R^2^ = 0.999, proving the detection capability of the pH sensor based on the test strip (Figure 3D).

### 3.3. Measurement of Glucose and pH in Artificial Sweat

**Figure 4:**
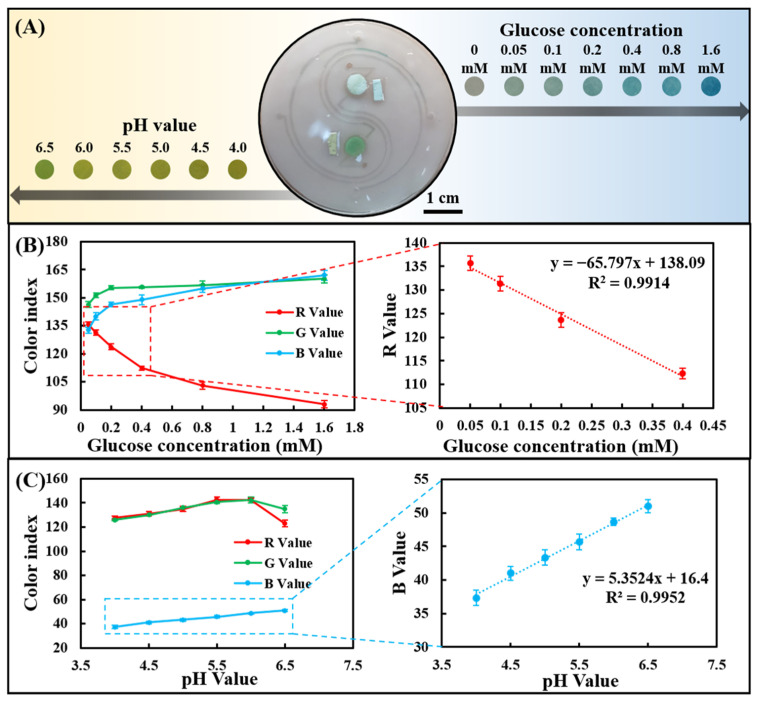
Determine the sweat glucose concentration and the pH value detection range in the chip using artificial sweat samples. (**A**) Optical images of the chip after reacting with artificial sweat samples. (**B**) RGB color index of the chip-based glucose assay and linear calibration curve for glucose in terms of the R-value. (**C**) RGB color index of the chip-based pH assay and linear calibration curve for pH in terms of the B value.

The channel layer, cover layer, and paper-based colorimetric sensor were plasma-treated and bonded to form a sweat chip for sweat analysis. Since the chip can be saturated with a very small amount of water, all tests were performed at saturation. The artificial sweat samples containing different concentrations of glucose were dropped on the injection port of the chip to evaluate the sensing performance of sweat glucose. As the glucose concentration increased from 0 to 1.60 mM, a clear color change could be observed in the images (Figure 4A). R, G, and B values in optical color images of glucose concentrations from 0 to 1.60 mM were analyzed using smartphone-based color analysis software. The R-value showed the most significant change compared with the G and B values (Figure 4B), which is consistent with the findings in Figure 3A. In addition, in the range of 0.05–0.40 mM, there was a good linear relationship between the R-value and low concentration of sweat glucose.

Likewise, artificial sweat samples with different pH values were dropped on the injection port of the chip to evaluate its sensing performance for sweat pH value. As the pH was increased from 4.0 to 6.5, a change in image color from yellow to greenish-yellow could be seen (Figure 4A). Color analysis software analyzes R, G, and B values in optical color images of sweat pH values ranging from 4.0 to 6.5. The B value showed a linear change compared to the R and G values (Figure 4C), in agreement with the results in Figure 3C. The B value showed a very good linear relationship between pH = 4.0 and 6.5, indicating that the chip can well monitor the dynamic changes of human sweat pH (4.5–6.5).

### 3.4. Dynamic Tracking of Glucose Concentration and pH in Human Epidermal Sweat

**Figure 5:**
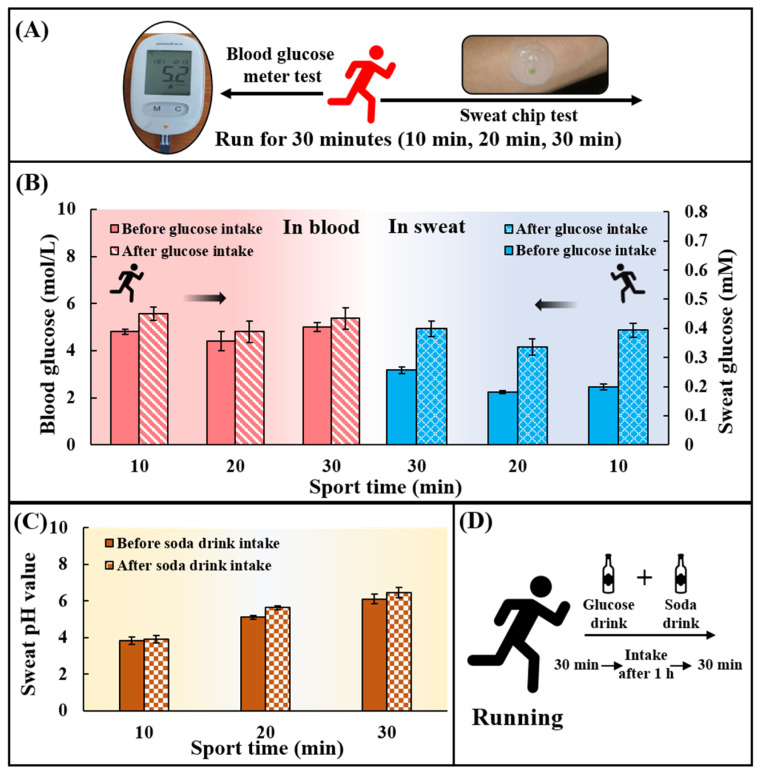
Practical application of the chip for sweat glucose and pH detection during exercise. (**A**) Blood glucose levels and sweat glucose concentrations were detected at 10, 20, and 30 min during exercise using a blood glucose meter and a sweat chip. (**B**) Changes in blood glucose and sweat glucose concentrations at 10, 20, and 30 min during exercise. (**C**) Changes of sweat pH value at 10, 20 and 30 min during exercise. There are two phases: before consuming the soda drink and after consuming the soda drink. (**D**) Replenishment of sports drinks. 250 mL of a drink containing 50 g of glucose was taken together with 375 mL of a soda drink with pH = 7.8 ± 0.4.

To verify the feasibility of using chips for in situ analysis of human epidermal sweat, healthy subjects (With a mean age of 22 years, a weight range of 80.0 ± 2.0 kg, and a height range of 180.0 ± 1.5 cm) ingested a high-carbohydrate and low-fat diet before the test to avoid the participation of fat in the energy supply during exercise. Subjects rested for 1 h after eating and then ran for 30 min. At 10, 20, and 30 min during exercise, the chip was used to collect sweat on the inside of the arm to detect the sweat glucose, and the blood glucose meter was used to collect the fresh peripheral blood from fingertips to detect the blood glucose concentration at the meantime (Figure 5A). After 30 min of running, subjects were supplemented with 250 mL of 0.20 g/mL glucose solution and 375 mL of soda drink with pH = 7.8 ± 0.4 (Figure 5D). After resting for 1 h, subjects ran for 30 min again, and the sweat glucose concentration and the blood glucose concentration were detected at 10, 20, and 30 min. It could be found that the sweat glucose concentration and the blood glucose concentration first decreased and then increased during exercise (Figure 5B). The participation of blood glucose in the energy supply during exercise led to a decrease in blood glucose concentration, and subsequent hepatic glycogenolysis caused an increase in blood glucose concentration. The change in blood glucose concentration affected the change in sweat glucose concentration. After ingestion of sports supplements, sweat glucose levels in all subjects were higher than before sports drink intake, but still within the normal range. By comparing blood and sweat, it could be concluded that although there were two orders of magnitude differences in glucose concentration between sweat and blood, both had the same change trend. It could also be seen that the pH value of all individuals after ingesting supplements was greater than that before ingesting supplements (Figure 5C). The tendency for the pH of sweat to increase during exercise may be due to the fact that prolonged exercise time caused a large amount of HCO^3−^ to accumulate on the surface of the skin, thereby increasing the pH of sweat. In conclusion, the in-situ analysis of human epidermal sweat at different times verified the ability of the microfluidic sweat chip to track human glucose concentration and pH value dynamically.

## 4. Conclusions

A wearable flexible microfluidic sweat chip was proposed in this work. Combined with a smartphone optical color recognition software, the chip can be used for non-invasive and immediate in situ detection of glucose concentration (0.05–0.40 mM) and pH (4.0–6.5) in epidermal sweat of the exercising population. This work optimized and constructed the paper-based sensor for sweat glucose and pH value detection based on the simple, reliable, and extremely low-cost H_2_O_2_ and pH test paper. The chip adopted a simple and reliable manufacturing process, which can be easily mass-produced and applied to meet the needs of most sports scenarios. The proposed microfluidic sweat chip can provide instant and reliable information on the physiological state of the body for exercise training monitoring. Unfortunately, high concentration ranges of sweat glucose and pH cannot be accurately detected by our proposed detection assy. Due to the limitations of our study, the chip cannot be applied in short-distance movement projects. At the same time, we did not include diabetes in the study, which we will focus on in the next study. In the future, by continuously optimizing the chip structure, exploring new physiological index detection methods and breaking through the bottleneck of the high-concentration detection of related substances, the microfluidic sweat chip can better help the development of the field of sports science.

## Data Availability

All data are presented in the main text of this manuscript.

## Supplementary Materials

The following supporting information can be downloaded at: https://www.mdpi.com/article/10.3390/bios13020157/s1, Figure S1: The fabrication processing of microfluidic sweat chip; Figure S2: The detailed chip design parameters; Figure S3: Paper-based glucose and pH sensors fabricated process; Figure S4: Microfluidic sweat chip chips are attached to different parts of the human body (forehead, arm, chest, etc.); Video S1: Using blue ink to test the water absorption performance of the microfluidic sweat chip.
