# Supplementary material for: Wearable Microfluidic Sweat Chip for Detection of Sweat Glucose and pH in Long-Distance Running Exercise
Source: Biosensors (Basel). 2023 Jan 19;13(2):157. doi: 10.3390/bios13020157 (PMC9953063; doi:10.3390/bios13020157)
Supplement: Supplementary file 1 [file biosensors-13-00157-s001.zip › biosensors-2105167-supplementary-done.pdf]

biosensors

MDPI

Supporting Information

Wearable Microfluidic Sweat Chip for Detection of Sweat Glucose and pH in Long-Distance Running Exercise

Dong Liu [1], Zhenyu Liu [2], Shilun Feng [3], Zehang Gao [3,4], Ran Chen [1,5], Gaozhe Cai [3,*] and Shengtai Bian [1,*]

[1] Microfluidics Research & Innovation Laboratory, School of Sport Science, Beijing Sport University, Beijing 100084, China
[2] School of Kinesiology, Shanghai University of Sport, Shanghai 200438, China
[3] State Key Laboratory of Transducer Technology, Shanghai Institute of Microsystem and Information Technology, Chinese Academy of Sciences, Shanghai 200050, China
[4] Department of Clinical Laboratory, Third Affiliated Hospital of Guangzhou Medical University, Guangzhou 510150, China
[5] School of Management, Beijing Sport University, Beijing 100084, China
* Correspondence: gaozhe@mail.sim.ac.cn (G.C.); bst13@tsinghua.org.cn (S.B.).

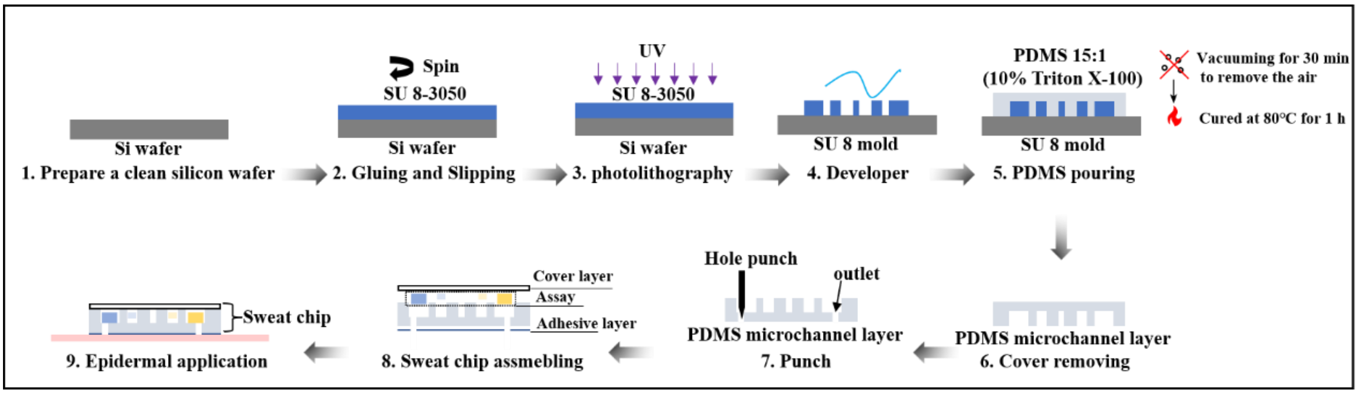

Figure S1. The fabrication processing of microfluidic sweat chip.

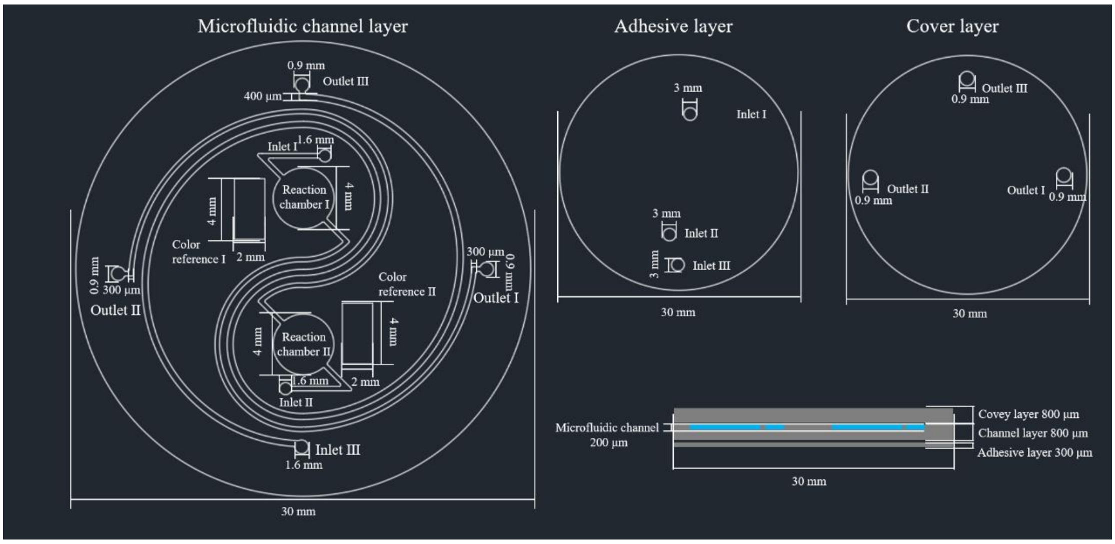

Figure S2. The detailed chip design parameters.

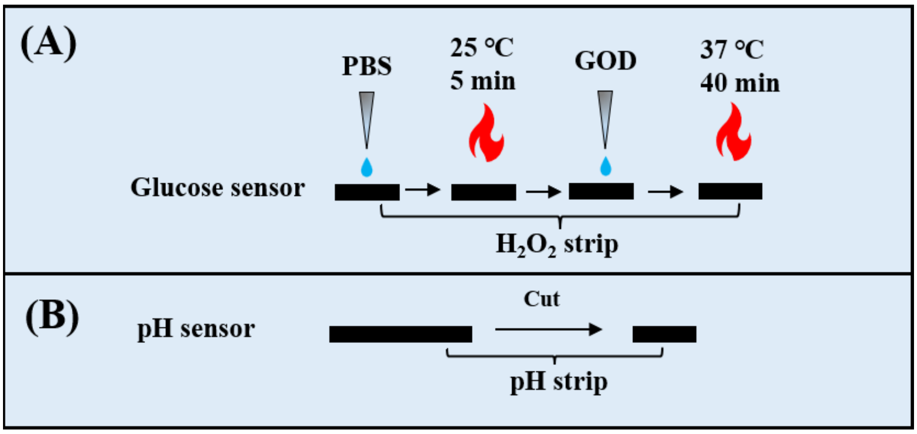

Figure S3. Paper-based glucose and pH sensors fabricated process. (A) The fabrication process of the glucose sensor is as follows: First, cut the H_2O_2 test strip into small discs (diameter: 3 mm). 2.5 μL of pH=5.7 PBS solution was dropped on the test paper and dried at 25°C for 10 min. Then, 2.5 μL of 200 U/mL GOD solution prepared with pH=5.7 PBS solution was dropped on the paper and dried at 37°C for 40 min. The functionalized glucose sensor was stored at 4°C for later use. (B) The production of pH sensor only needs to cut the pH test paper into 3 mm diameter discs for later use, which is very convenient.

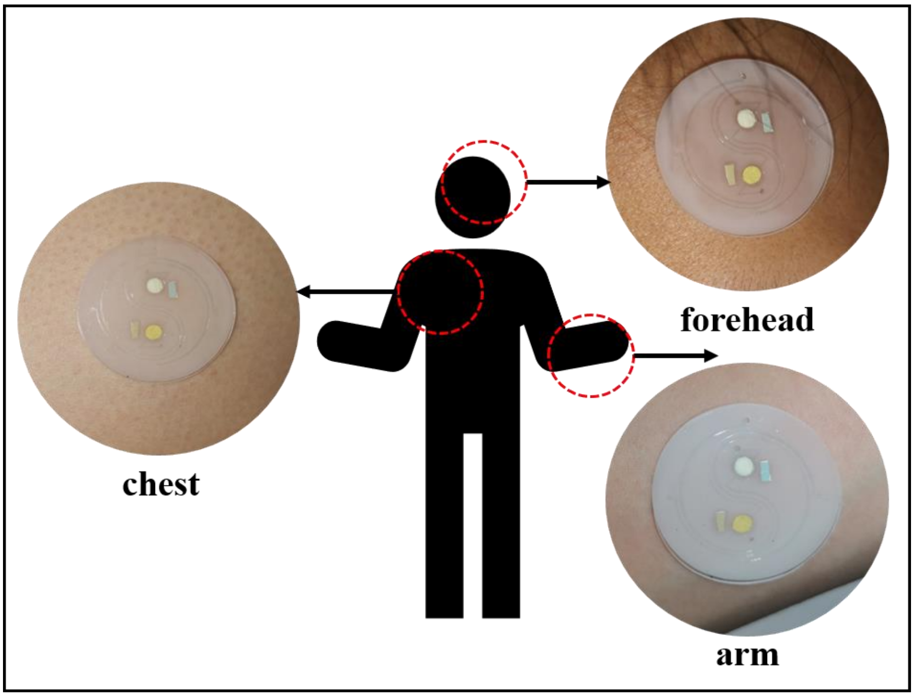

Figure S4. Microfluidic sweat chip chips are attached to different parts of the human body (forehead, arm, chest, etc.).

Test of water absorption perfo

Video S1: Using blue ink to test the water absorption performance of the microfluidic sweat chip.
